# Single step phase optimisation for coherent beam combination using deep learning

**DOI:** 10.1038/s41598-022-09172-2

**Published:** 2022-03-25

**Authors:** Ben Mills, James A. Grant-Jacob, Matthew Praeger, Robert W. Eason, Johan Nilsson, Michalis N. Zervas

**Affiliations:** Optoelectronics Research Centre, University of Southampton, Southampton, SO171BJ UK

**Keywords:** Applied optics, Optical techniques, Fibre lasers

## Abstract

Coherent beam combination of multiple fibres can be used to overcome limitations such as the power handling capability of single fibre configurations. In such a scheme, the focal intensity profile is critically dependent upon the relative phase of each fibre and so precise control over the phase of each fibre channel is essential. Determining the required phase compensations from the focal intensity profile alone (as measured via a camera) is extremely challenging with a large number of fibres as the phase information is obfuscated. Whilst iterative methods exist for phase retrieval, in practice, due to phase noise within a fibre laser amplification system, a single step process with computational time on the scale of milliseconds is needed. Here, we show how a neural network can be used to identify the phases of each fibre from the focal intensity profile, in a single step of ~ 10 ms, for a simulated 3-ring hexagonal close-packed arrangement, containing 19 separate fibres and subsequently how this enables bespoke beam shaping. In addition, we show that deep learning can be used to determine whether a desired intensity profile is physically possible within the simulation. This, coupled with the demonstrated resilience against simulated experimental noise, indicates a strong potential for the application of deep learning for coherent beam combination.

## Introduction

High-power fibre lasers^1,2^ have seen increasing numbers of applications over the past few decades, as their maximum output power has risen by orders of magnitude during this time^3,4^. Advancing beyond kilowatt average power levels, however, is challenging since high-power fibre lasers generally operate with a small mode field size and large propagation length, and hence, are subject to a range of nonlinear effects, such as Stimulated Raman Scattering^5^, Stimulated Brillouin Scattering^6^, and the optical Kerr effect^7^. Although the mode area can be increased, this reduces the threshold for transverse mode instability. An increasingly important technique for enhancing the total average power without increasing the average power of a single fibre is through the combination of multiple fibres emitting spectrally narrow co-aligned colinearly polarised beams, generally known as coherent beam combination^8–11^. However, an important consequence of combining multiple fibres is that changes in their relative phase will modify the resultant spatial intensity profile. Therefore, random differential phase fluctuations that are introduced externally or are intrinsic to the fibre laser amplification process, which might not be problematic when using a single fibre, are now a fundamentally limiting complication. There is therefore great interest in devising methods for identifying and correcting the phase of each fibre, in real-time, during operation. In practice, where the phase of each fibre can be accurately identified, a correcting signal could be sent to the phase actuator associated with each fibre, hence, resulting in a corrected focussed intensity profile.

Whilst there are methods for identification of the fibre phases through spatial interference or temporal beating^12,13^, a potentially more robust and lower cost solution is possible via analysis of the focal intensity profile. An added attraction of this is that it works directly with the targeted property, i.e., a desired beam profile. In practice, the focal intensity profile could be recorded on a camera, through using a beam splitter after the fibre array and focussing lens, on a separate beam path to that used for materials processing. The disadvantage of this relatively simple approach would be that the recorded focal intensity profile cannot directly provide information regarding the phase of each fibre, as only the modulus-squared of the electric field is recorded. Iterative phase retrieval methods exist, such as Gerchberg–Saxton and hybrid input–output (HIO)^14^ algorithms. Other methods such as hill-climbing^15,16^ (e.g., stochastic parallel gradient descent, SPGD^17^) are often used, but require that the power is focused into a single spot. However, such methods are iterative, and in practice, a single-step, low-latency process would clearly be preferable.

Deep learning has recently seen a wide range of applications across laser optimisation^18,19^ and laser materials processing^20,21^, where neural networks have been shown to be as effective, or more effective, than traditional modelling approaches^22^. Critically, deep learning has the fundamentally important advantage of computation speed, as neural network implementations typically take just tens of milliseconds per calculation. Deep learning is particularly useful when the underlying processes are challenging, or impossible, to describe mathematically^23^, and hence, neural networks are a natural choice for solving the challenge of reconstructing phases from an intensity profile, as shown in the general area of coherent diffractive imaging^24,25^.

Deep learning has also started to be applied to coherent beam combination. Recent results in this field include the experimental demonstration of coherent control of 107 beams^10^ and a 7-channel 7 kW combination^11^ using stochastic gradient descent, which is a gradient-based search method used here for maximising the power transmitted through an aperture at the focal plane. Subsequent improvement to this approach involved the application of momentum to the algorithm^26^. Whilst stochastic gradient descent is an iterative approach, the rapid iteration speed (typically > 1 MHz) offers a strong compensation. However, the capability of the approach generally decreases linearly as the number of fibres is increased^16^, with typically of order ten iterations needed per fibre used. In addition, stochastic gradient descent does not target specific fibres for optimisation, and it introduces random intensity noise fluctuations due to the dithering process required for solution discovery. There is therefore great interest in applying neural networks to the spatial intensity distribution (rather than total intensity) that can be recorded on a camera, at or near the focal plane, for achieving single step phase identification and correction.

However, this unleashes the fundamental challenge of recovering the phase profile from a spatial intensity profile, where there may be many (or even infinite) phase profiles that can result in a specific spatial intensity profile^27^. There have been many variants of solutions to this challenge in the field of coherent beam combination. Chang et al.^28^ demonstrated that interference with a reference beam can allow for identification of the fibre phases. Wang et al.^29^ showed that a diffractive optical element can produce a set of interference patterns that can enable phase identification, but that due to the cyclical nature of the interference fringes, the neural network struggled to operate over the full range of phase values (although the authors did show that the approach always converged, and that small phase errors could be solved in a single step). Hou et al.^30^ discussed again the non-uniqueness between the focal intensity profile and the phase profile with regard to coherent beam combination and proposed using a neural network for phase identification using a camera positioned away from the focal plane. This neural network solution was then improved further by stochastic gradient descent using a camera at the focal plane. Hou et al.^31^ later showed that this approach can also be applied for the creation of orbital angular momentum beams, when using a ring of fibres. There have also been several key results in the field of deep reinforcement learning applied to coherent beam combination^32,33^, which is a technique that allows a neural network to learn by trial and error on the experimental setup or in a virtual environment^34^.

A clear theme in the literature at the interface of deep learning and coherent beam control is therefore the challenge of the non-uniqueness between a focal intensity profile and the associated phase profile of the fibres. Here, in this manuscript, we show that by setting up the phase identification challenge as an image-to-image ‘phase retrieval’ problem (i.e. where the neural network transforms an intensity profile image into a phase profile image), we are able to apply two constraints that reduce the complexity and the non-uniqueness of this challenge. Firstly, we set the phase of the central fibre to zero, hence removing the possibility of a global phase offset, and hence the neural network identifies the phase of each fibre relative to the central fibre. Secondly, through using a 2D array (i.e. an image) for the reconstructed phases, the fibre positions are fixed and hence a compensatory linear phase gradient across the predicted fibre array is not a possible solution. These two constraints allow us to train a neural network that can identify the fibre phases in a single step without the need for additional experimental constraints, such as introducing interference effects between the fibres, artificially restricting the range of possible phases, or observing the intensity profile away from the focal plane. We then show that bespoke beam shaping can be achieved in the same single step, through an additional static phase correction. Finally, we demonstrate that by combining two neural networks, we can offer a single step method for solving the well-known problem of verifying whether a specific (i.e. desired) focal intensity profile is possible with a given spatial arrangement of fibres.

Whilst both coherent beam combination and bespoke beam shaping are discussed here, it is important to realise they have fundamentally different applications. In general, the primary objective of coherent beam combination is to overcome the power scaling limit of a single fibre and unlock the capability for considerably higher directed laser power. Alongside unlocking novel manufacturing capabilities, applications here are often based around grand visions such as space debris removal^35^ and particle acceleration^36^. Whilst the objective for bespoke beam shaping may also include maximising power delivery, it is generally more strongly aligned to the generation of exotic intensity profiles^37,38^, with associated applications including telecommunications^39^ and manufacturing^40^. In this manuscript both techniques are included, as we show that achieving coherent beam combination also offers the potential for subsequent bespoke beam shaping, all as a single process when using deep learning.

## Results

The primary objective of this work was to demonstrate a method for the identification of the phase profile in a fibre array, when only observing the intensity profile at, or near, the focus. Fundamentally, the challenge of phase identification arises due to the nature of measuring the intensity profile, where the phase information is obfuscated as only the intensity associated with the electric field is recorded. Therefore, whilst the transformation from known intensity and phase for each fibre, to the resultant focussed intensity profile is mathematically trivial, the inverse transformation from a measured intensity profile back to the intensity and phase of individual fibre phases generally requires a more complex approach, such as iterative algorithms (even when each fibre intensity is assumed to be constant). In this work, we demonstrate that by setting up the challenge of the identification of the phase of each fibre as a two-dimensional phase retrieval problem, we can identify an accurate solution, in a single step, using deep learning.

**Figure 1:**
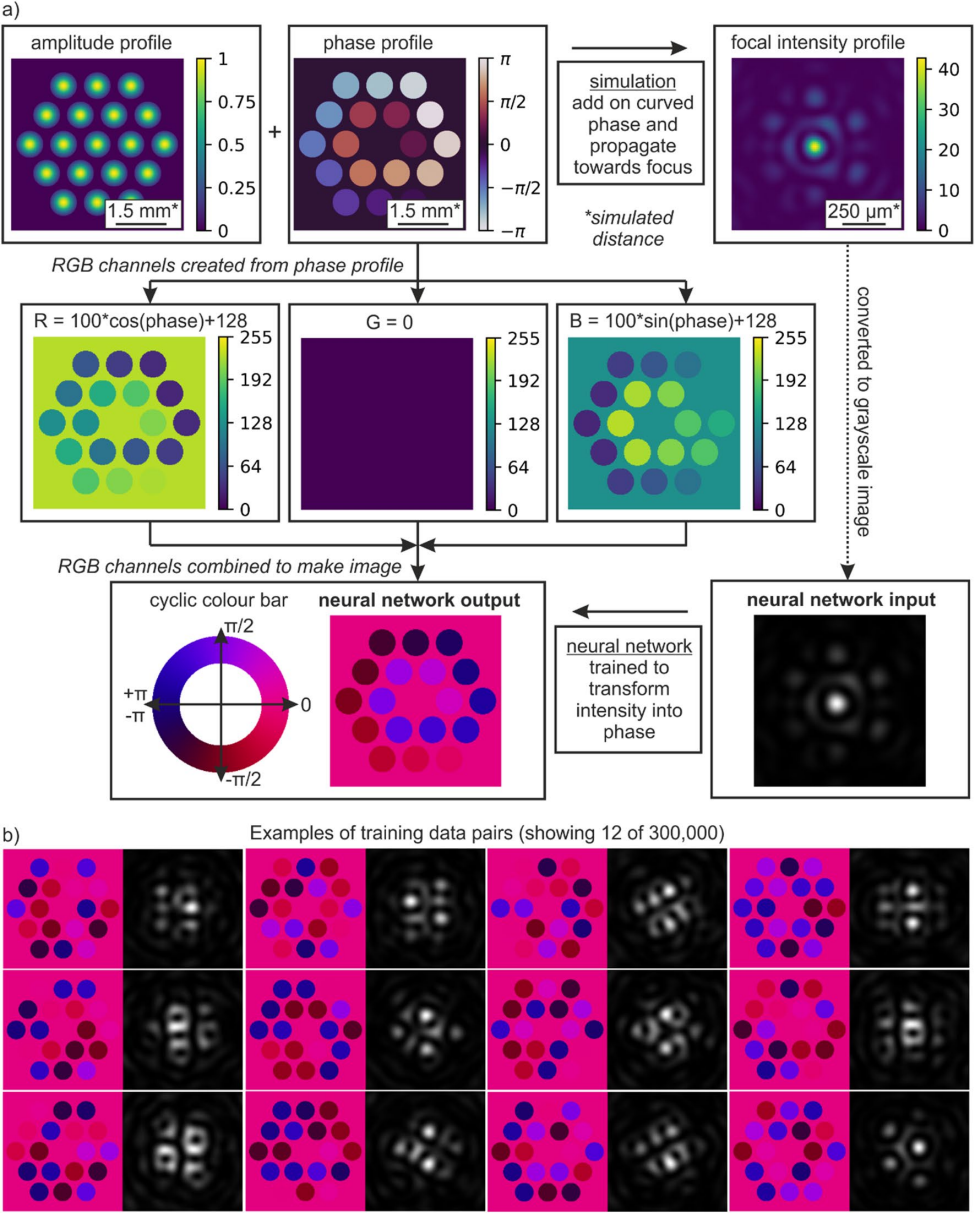
Process for creation of training data suitable for training a neural network to transform a focal intensity profile into the associated phase profile that shows the phase of each fibre. Showing (**a**) schematic of application of beam propagation simulation for creating neural network training data, which produces a 256 × 256 intensity image and associated 256 × 256 phase image. Showing (**b**) twelve examples of training data pairs.

Here, the deep learning approach uses a conditional generative adversarial network^41^ that can transform an image associated with one domain into an image associated with another domain. We therefore use the neural network for transforming spatial intensity profiles into spatial phase profiles, hence acting as a single step phase retrieval calculator. Whilst deep learning eliminates the need for a physical understanding of a transformation, in practice the scientific effort is transferred to the production of suitable training data. The method for creating training data for this work is shown in Fig. 1, where randomly generated phases and a fixed intensity profile are applied to nineteen fibres formed into a hexagonal close-packed array, and the associated focused intensity profile is calculated via beam propagation mathematics^42^ with the addition of a curved phase to simulate the effect of the focussing lens. As illustrated in the figure, a notable adaptation was to describe the phase using sine and cosine functions in two separate colour channels, to provide a cyclical colour change associated with the phase. A set of twelve randomly chosen training pairs are shown in the inset (b) of the figure. A total of 3 × 10^5^ training pairs and 1500 testing pairs were created.

Figure 1a shows a flowchart that expresses the simulation process for creating training data for the neural network. Firstly, an array is created that describes the amplitude profile (“amplitude profile”), where the fibres are arranged in a hexagonal close-packed array, and the output of each fibre has a Gaussian profile (see “Methods” section for specific details on beam profile). Phase information is then created (“phase profile”), where the phase value for each fibre is randomly chosen between − π and + π, except for the central fibre, which always has a phase of 0. The amplitude and phase are combined to produce the electric field immediately after the exit plane of the fibres. A spherical phase profile is then added to this electric field, to simulate the application of a focussing optic. The light is then propagated towards the focus, and the intensity of the electric field is recorded on a simulated camera (“focal intensity profile”). The flowchart then shows the process for creating training data for the neural network input from the focal intensity profile, and for the neural network output from the phase profile. For this work, the well-established neural network known as “pix2pix”^41^ was used (see “Methods” section for technical details), which can be used to transform a 24-bit RGB image into another 24-bit RGB image. The neural network input and outputs were therefore formed as 24-bit RGB images, where the pixels in each colour channel (i.e. each of the RGB channels) could take any integer value between 0 and 255 inclusive (i.e. 8-bit per colour channel).

For creating the neural network output corresponding to the phase profile, two of the three colour channels were used. This allowed the phase values to be represented by a cosine and sine transformation, which ensured a cyclical continuity between the phase values of -π and + π. The R (red) and B (blue) channels were transformed using cosine and sine functions respectively, and the G (green) channel was set to 0. When these R, G and B channels were combined, a single image was produced (“neural network output”), where the conversion from angle to colour is shown (“cyclic colour bar”). For creating the neural network input, the focal intensity profile was used for each of the R, G, and B channels, hence converting the simulated focal intensity profile (“focal intensity profile”) into a grayscale image (“neural network input”). The neural network was then trained (using generated data pairs such as those shown in Fig. 1b to convert the image corresponding to the “neural network input” into the image corresponding to the “neural network output”. In other words, the neural network was trained to transform a simulated focal intensity profile into the associated simulated phase profile, hence learning the capability for making a prediction of the phases for any given focal intensity profile.

As multiple spatial phase images can result in the same focal intensity, the task for the neural network of predicting phase information from a focal intensity profile would be one-to-many. In general, neural networks are designed for learning one-to-one mappings, and hence will only produce a single output for any given input. An important challenge here was therefore to define an additional set of constraints to transform the one-to-many problem into a one-to-one problem. The first constraint was to set the phase of the central fibre to zero in all cases, as this removed the effect whereby adding a constant phase value to all fibres would produce an identical focal intensity profile. Consequently, the neural network would predict the relative phase between each fibre and the central fibre. The second constraint was to fix the positions of the fibres in the phase profile image, as this removed the effect whereby a phase image that is spatially shifted but combined with a linear phase gradient could produce an identical focal intensity profile. The motivation for identification of the phase profile of the fibre array from the intensity profile is for demonstrating the concept of firstly phase correction, and secondly bespoke beam shaping. Here, we do not discuss the engineering challenges, such as beam alignment, associated with phase control of multiple individual optical fibres, and instead our focus is the concept of phase identification and correction.

**Figure 2:**
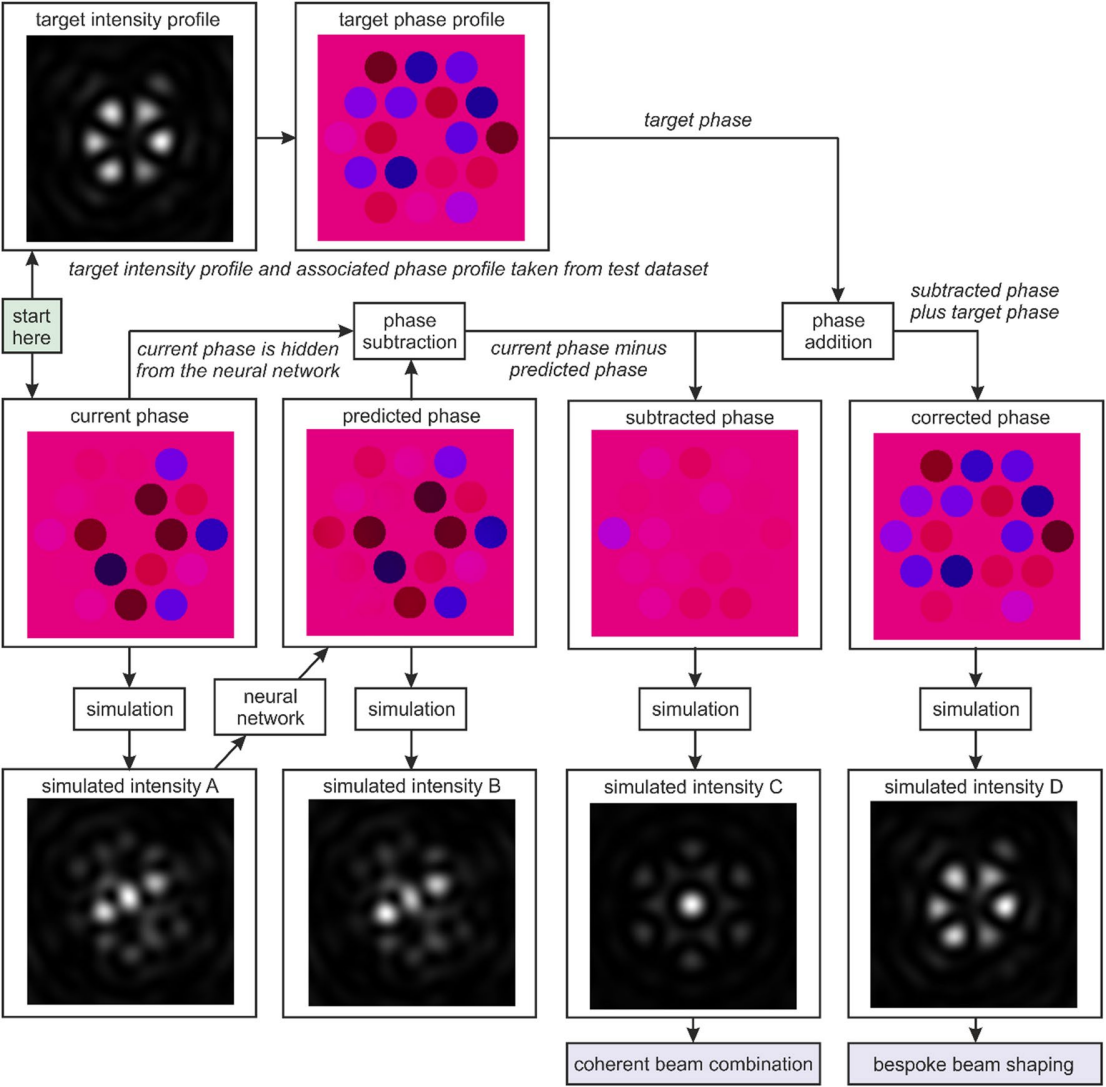
Application of a neural network for bespoke beam shaping for any phase that is unknown to the network. Starting from a current phase (which is unknown to the neural network), the associated simulated intensity is processed by the neural network and the phase is predicted. Subtracting the predicted phase profile from the (hidden) current phase profile produces a flat phase, with error depending on the prediction accuracy. At the same time, the phase profile for a desired intensity profile can be added to the corrected phase, to produce the desired intensity profile. This whole process is possible without knowledge of the current phase and could be completed in a single step.

Figure 2 shows a flowchart that explains the process of neural network identification of phases, and subsequently demonstrates a potential technique for coherent beam combination and bespoke beam shaping. The process can be explained by starting at the “start here” box and following downwards to the randomly chosen phase profile (“current phase”), which is both unknown to the neural network in this flowchart process and was not used during the training process. In practice, the “current phase” would represent the phase profile that is occurring at this point of time on the experimental setup, but where this phase profile is of course unknown to the experimenter. The current phase is then propagated to the simulated focal plane and the intensity recorded (“simulated intensity A”). This intensity profile is used as the neural network input, which then predicts a phase profile (“predicted phase”). For interest, in this flowchart the “predicted phase” is also simulated to the focal plane (“simulated intensity B”) for comparison with the focal intensity profile corresponding to the correct phase profile (i.e. “simulated intensity A”). In the flowchart, the “current phase” and “predicted phase” are similar, and their associated focal intensity profiles (i.e. simulated intensity A and B) are also similar, indicating the accuracy of the neural network phase prediction. The “predicted phase” can then be subtracted from the “current phase”, which in practice could be achieved using phase controllers associated with each individual fibre, yielding the “subtracted phase”. As seen by “simulated intensity C”, which shows a high intensity in the central diffraction order, the “subtracted phase” could be used for coherent beam combination. At this stage, the application of bespoke beam shaping can be introduced. From the initial “start here” box, and following upwards to the “target intensity profile” and the associated “target phase profile”, it is possible to see how the addition of a custom phase to the “subtracted phase” profile can produce the bespoke phase profile (“corrected phase”) and the associated bespoke intensity profile (“simulated intensity D”). Note the similarity between the “simulated intensity D” and the “target intensity profile”. In summary, despite starting from an unknown initial phase profile (i.e. “current phase”) the application of a neural network is shown here to enable both coherent beam combination and bespoke beam shaping in a single process.

As shown in Fig. 2, the neural network is capable of the identification of the phase profile from the intensity profile (note the similarity between the current i.e. actual, and predicted phase profiles). When this predicted phase is subtracted from the current phase (which is both random and unknown to the neural network), the result is the creation of a flat phase profile (labelled subtracted phase). In some applications the subtracted phase may offer the desired output, however, this can optionally be augmented with a bespoke phase profile (i.e. the target phase can be added in order to produce the corrected phase output). In practice, one could envisage that this phase subtraction and phase addition could be managed in a single step via direct control of the phase actuator of each fibre. Whilst the figure shows the transformation into a sixfold ring intensity profile, in practice, the end result could be any physically possible spatial intensity profile.

Due to diffraction effects, the focal intensity profile from hexagonal close-packed arrays of 7 and 19 fibres can have a similar appearance, as generally the phases of the central 7 fibres determine the larger-scale features and the phases of the outer 12 fibres determine the smaller-scale features (see^43^ for a detailed discussion). One might therefore expect the phases of the outer 12 fibres to be predicted less accurately by the neural network. This is indeed observed, as the standard deviation of the neural network prediction errors for the inner 7 and outer 12 fibre phases were 0.076π and 0.13π respectively. The standard deviation of a random guess of the phase would be $2\pi /\sqrt {12}$ or 0.57π, hence confirming that the neural network was able to identify features in the simulated focal intensity profiles corresponding to the outer 12 fibres, which therefore confirms that the outer 12 fibres did indeed contribute to the structure of the focal intensity profiles.

## Discussion

Whilst here the neural network was provided with training data that corresponded to a theoretically perfect simulation, the resilience of the neural network to noise within the intensity profiles (i.e. camera images) is important to quantify. For this work, we introduced a unit of simulated experimental noise, which corresponded to a normal distribution with mean zero and standard deviation as the square root of the magnitude of the intensity profile, and which was chosen to be a realistic model of noise one might observe experimentally from a camera. As the simulated intensity profiles were converted to a grayscale 8-bit image file for use with the neural network (see Fig. 1), the possible intensity values for each image pixel varied between 0 and 255. Therefore, for one unit of experimental noise, a pixel value of 100 would generally vary between 90 and 110 (i.e. approximately 68% of values would fall in this range). The neural network was tested with 1, 10 and 100 units of simulated experimental noise, and a comparison made of the predictive accuracy.

**Figure 3:**
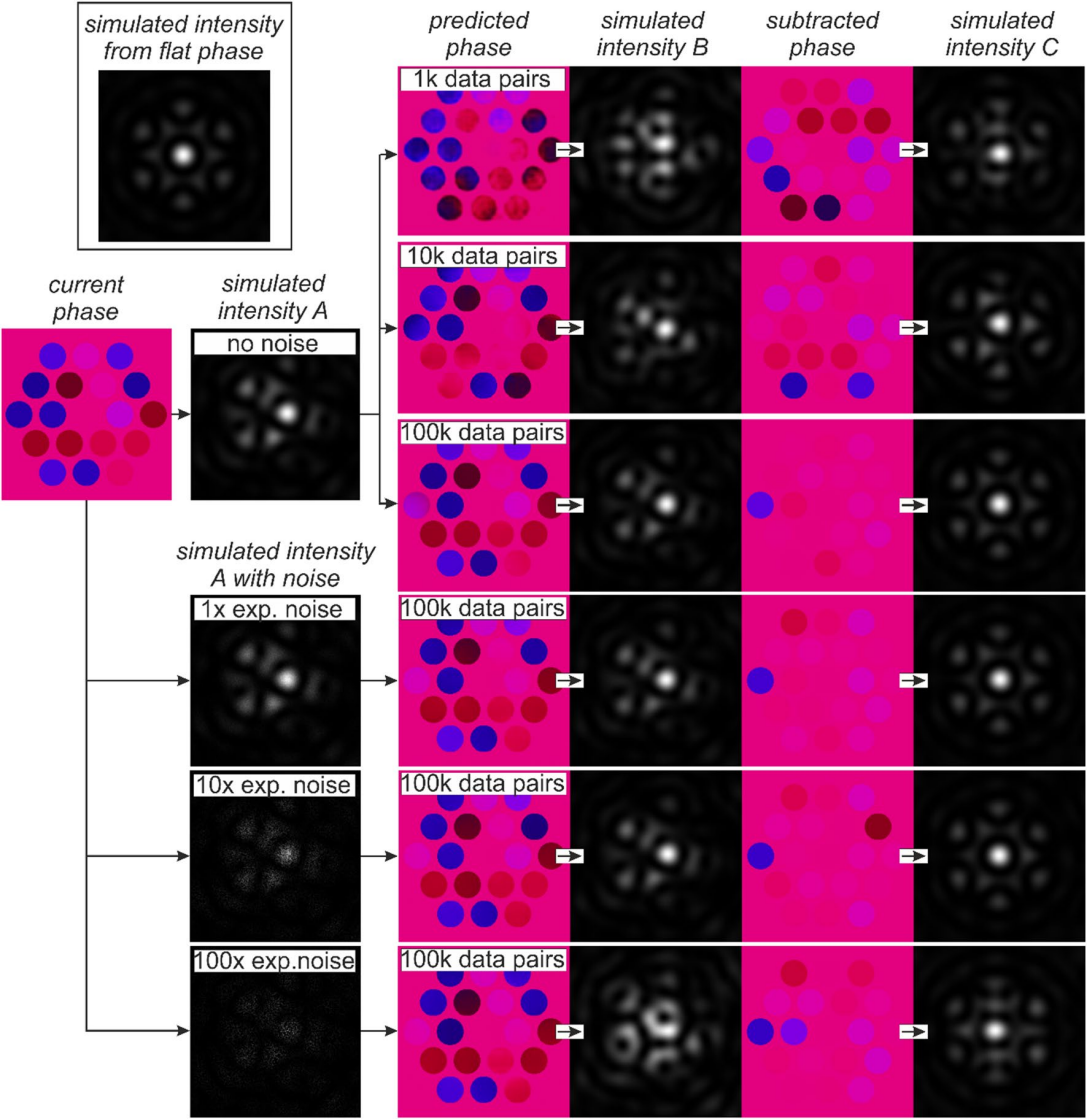
Neural network capability for different numbers of training pairs, and under different levels of simulated experimental noise. The results show clear improvements in the predictive capability as the number of training pairs is increased. The neural network is shown to be resilient under noise conditions that might be experimentally observed. Also shown, for comparison, is the intensity profile associated with a flat phase.

Figure 3 shows a flowchart that explains the process for investigating the resilience of the neural network to simulated experimental noise when making a phase prediction from a focal intensity profile. The flowchart also shows the sensitivity of the neural network accuracy to the number of training data pairs. In the top left of the flowchart, the intensity profile corresponding to a flat phase (“simulated intensity from flat phase”) is presented as a reference. Starting from the initial and unknown phase (“current phase”), the associated focal intensity profiles are calculated with no noise (“simulated intensity A”) and with different levels of noise (“simulated intensity A with noise”). The first column (“predicted phase”) shows the neural network phase prediction for 1 k, 10 k and 100 k training pairs with no noise, and the prediction for 100 k training pairs with 1 ×, 10 × and 100 × simulated experimental noise. The predicted phases in this column can therefore be compared with the “current phase”. The second column (“simulated intensity B”) shows the simulated focal intensity profiles associated with the predicted phases, where each intensity profile can be compared to “simulated intensity A”. The third column (“subtracted phase”) shows the difference between the “current phase” and the “predicted phase”, which in practice could be achieved using phase controllers associated with each individual fibre. The fourth column (“simulated intensity C”) shows the simulated focal intensity associated with the “subtracted phases”, where each intensity profile can be compared to the focal intensity profile associated with a flat phase (“simulated intensity from flat phase”). The flowchart shows clear improvements in the neural network prediction accuracy as the number of training pairs is increased up to 100 k, and the neural network is shown to be resilient to simulated experimental noise (i.e. “1 × exp. noise”).

As can be seen in Fig. 3, the effect of noise at 1 and 10 units was minimal. The observation that the neural network was robust against a level of noise that could be typical in an experiment is perhaps surprising, given that the network was only trained on simulations without noise. Also shown in Fig. 3, are examples of predicted phase profiles for different numbers of training data pairs. There is a clear improvement in predictive capability as the number of training pairs is increased.

**Figure 4:**
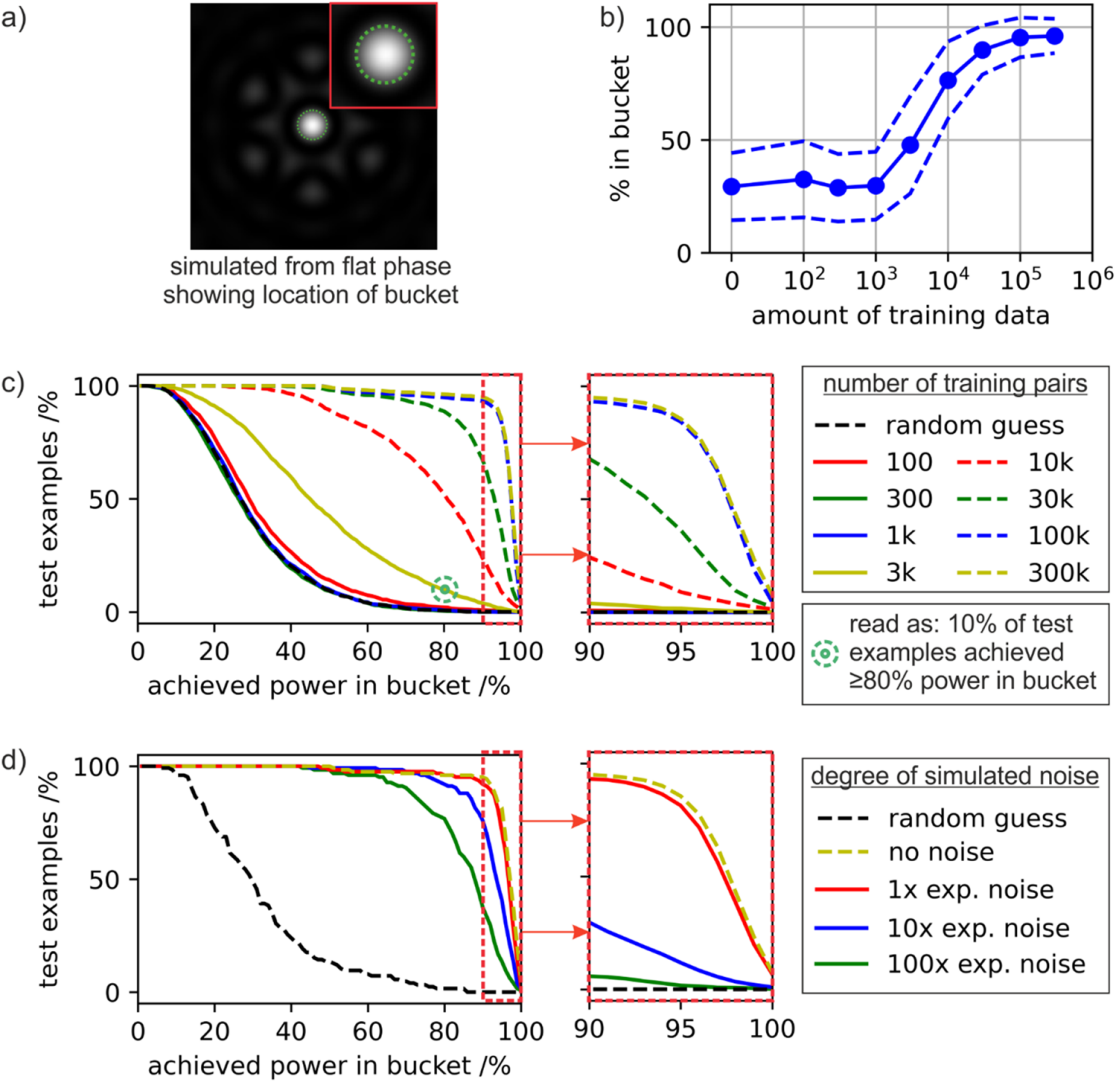
Analysis of errors for 1500 randomly chosen test examples showing (**a**) concept of power in the bucket, corresponding here to the percentage of intensity in the bucket for the corrected intensity profile divided by the percentage in the bucket for the flat phase case, (**b**) the mean and standard deviation of power in the bucket for different numbers of training data pairs, (**c**) distribution of test examples vs achieved power in the bucket for different amounts of training data and (**d**) distribution of test examples vs achieved power in the bucket for different levels of simulated noise for the 300 k case. Note that the line associated with the random guess is the same in both (**c**) and (**d**).

The effect of the amount of training data and degree of simulated noise on the neural network predictions are evaluated in more detail in Fig. 4, where predictions from 1500 test examples are presented. A test example is defined here as a pair of images corresponding to a randomly generated phase profile and the associated simulated focal intensity profile, where the pair of images was not used for training the neural network. A test example can therefore be used for quantifying the accuracy of the neural network in predicting the phase profile directly from a focal intensity profile.

A key metric here is achieved using an arbitrarily chosen boundary, generally referred to as a bucket, which corresponds in this case to the approximate size of the central spot when a flat phase profile is chosen. The power in the bucket, i.e. the intensity that is contained within the chosen region, can then be used to provide a quantitative comparison of the neural network predictive capability under different conditions. Here, the power in the bucket for any intensity profile is defined as the percentage of intensity contained within the bucket divided by the percentage of intensity contained within the bucket for a flat phase profile. Hence, in this work, the power in the bucket percentage metric is a measure of comparison with the “perfect” flat phase profile. Whilst the maximum bucket efficiency for a coherent beam combination simulation depends on a range of factors such as size of the bucket and the beam profile^44^, for the simulation used in this work when all fibres have equal phase value, the central diffraction order contains ~ 35% of the power and there is an associated 44 times increase in on-axis intensity. For comparison, when the phases are randomly generated there is a 10 ± 7 times increase in on-axis intensity.

Figure 4a shows the location of the bucket (green dashed line) used for evaluating the neural network accuracy, overlaid on the simulated focal intensity for a flat phase profile, where the inset (red square) shows the location in more detail. To quantify the accuracy of the neural network in predicting phase profiles, each predicted phase profile was subtracted from the true phase profile, and this subtracted phase profile was propagated towards the focal plane to produce a focal intensity profile, where the power in bucket percentage is calculated. This percentage value is then divided by the power in bucket percentage value associated with a flat phase profile, and hence, in Fig. 4, a power in bucket percentage value of 100% represents a perfect phase prediction.

Figure 4b shows the mean and standard deviation of power in bucket percentage, for all 1500 test examples, when the neural network is trained on different numbers of training data pairs. Figure 4c shows the percentage of test examples that achieved greater than a specific percentage of power in bucket (as defined on the horizontal axis), for a range of training data pairs, along with a random guess (i.e. using a random number generator to predict the phases of each fibre). The green circle-target shows an example to help understand the graph, corresponding to 10% of test examples achieving ≥ 80% power in bucket when the neural network had been trained using just 3 k data pairs. Figure 4d shows the cumulative distribution of percentage of test examples that achieved greater than the specified percentage of power in bucket, for varying degrees of simulated noise, along with a random guess. Both c) and d) show an expanded view of the right-hand side region of the graph.

The figure shows that for 10^3^ training pairs or fewer, the neural network predictive capability is no better than a random guess. Likewise, for more than 10^5^ training pairs, almost no further improvement is demonstrated. The cumulative distribution shows that increasing the amount of training data from 10^4^ to 10^5^ increases the percentage of test data that achieves 90% power in the bucket from 23 to 93%. This result implies that when applying deep learning to a phase retrieval problem, there may be a significant improvement in accuracy achieved through additional training data, but equally there may be a saturation point where returns diminish, and additional training data no longer improves the accuracy. The cumulative distribution associated with simulated experimental noise shows clearly that the neural network predictive capability is only slightly affected by the inclusion of noise, especially if the magnitude of that noise is relatively small.

**Figure 5:**
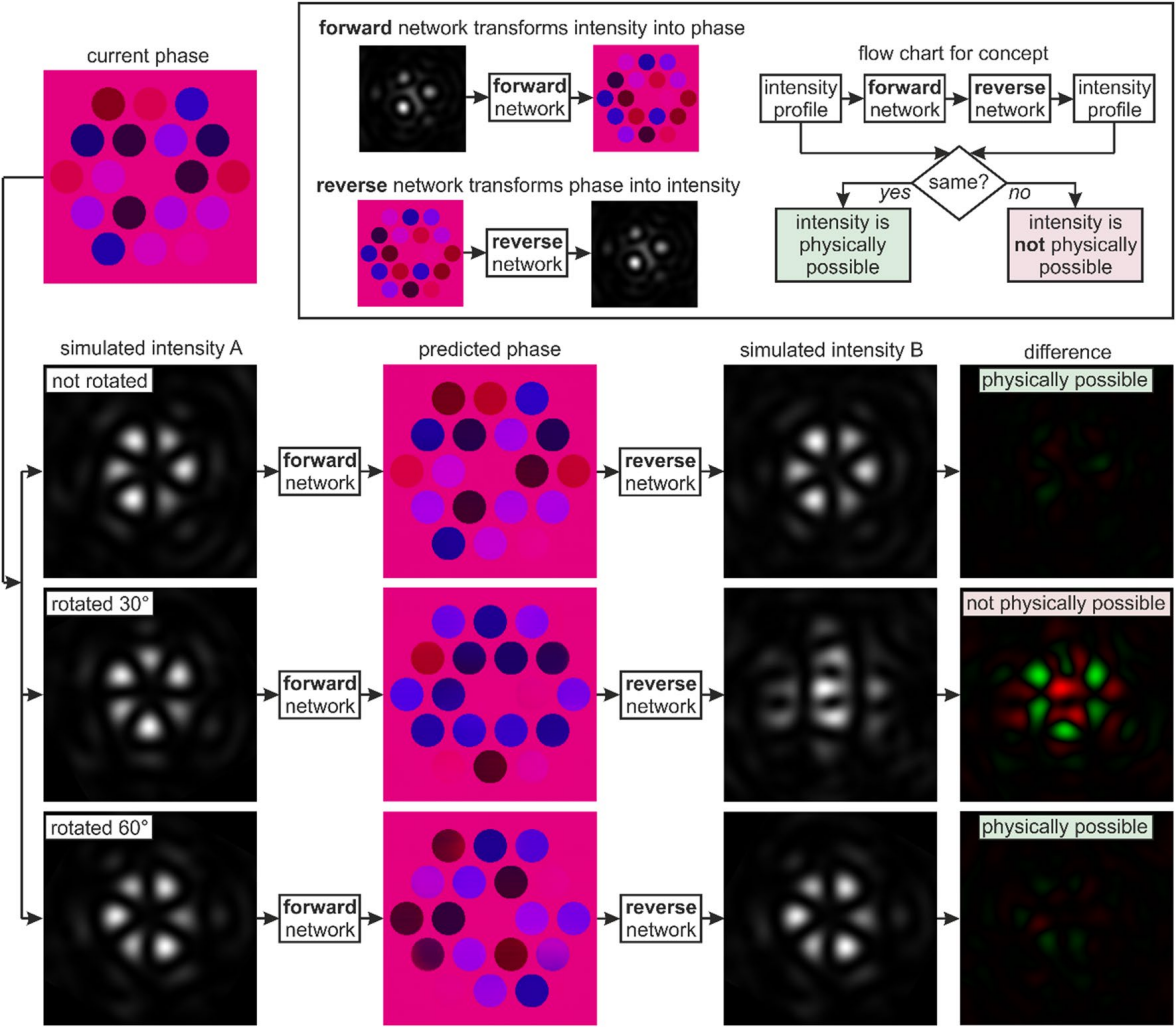
Concept of using a forward and reverse neural network, in combination, for single step identification of whether an intensity profile is physically possible. In this case, the approach identifies whether a particular intensity profile is possible in this simulation. This method is possible as all simulated phases lead to simulated intensities, but not all simulated intensities lead to simulated phases. Due to the sixfold rotational symmetry of the fibre array, a 30-degree rotation of the ring-shaped intensity profile is not a possible intensity profile, and hence there is no cyclicity between the domains (i.e. there is a difference between the input and output intensity profile). The difference column highlights where intensity is removed from the input intensity (green) or added to the input intensity (red), when comparing with the output intensity. The image pixel value for the red and green range from 0 to 255 according to the difference in pixel values for the input and output intensity profiles.

As illustrated earlier in Fig. 2, where a random intensity profile is modified into a ring pattern in a single step, a significant advantage of using an array of fibres is the prospect for real-time bespoke beam shaping. However, due to challenges associated with transforming an intensity profile into a phase profile, there is generally no direct way of knowing whether a desired intensity profile is physically possible. However, as shown in Fig. 5, through an innovative application of two neural networks, a single step process can be devised to test the validity of any desired intensity profile. To achieve this, a second neural network is trained that can perform the reverse operation, i.e. to transform a phase profile into an intensity profile. Whilst such a transformation is mathematically trivial, there is still a computational cost associated with this calculation, which can generally be reduced through the application of a neural network. To determine whether an intensity profile is physically possible (under the conditions simulated in this work), the intensity profile is passed through the first network, and the predicted phase profile is passed through the second network, which then predicts a second intensity profile. If the output of the second neural network is equal to the input of the first neural network, then the input intensity profile is possible. If the output intensity is different to the input intensity, then the input intensity is not possible. This effect can be understood by the relationship, within this simulation, where all possible phase profiles lead to an intensity profile, but not all intensity profiles lead to a phase profile (e.g. a square focussed intensity profile is not possible due to the fixed intensity distribution assumed at the fibre output). Using two neural networks in sequence is therefore a test of cyclic consistency between the two domains.

Figure 5 shows a flowchart that provides an example application of this technique, via the application of a focal intensity profile that corresponds to a sixfold ring. The starting point of the flowchart is the initial phase profile (“current phase”), which is then propagated to the focal intensity plane (“simulated intensity A”) using the beam propagation simulation. In this case, the “simulated intensity A” is shown three times, namely with a 0-degree rotation (“not rotated”), a 30-degree rotation, and a 60-degree rotation. Due to the sixfold rotational symmetry, the 0-degree and the 60-degree focal intensity profiles have the same alignment, but there are noticeable differences in the relative intensities of the interference peaks. Critical for this demonstration, the 30-degree rotation is not a physically possible focal intensity profile for the simulation used here, due to the fixed position of the fibres. This was also confirmed numerically through the generation of 500 k additional training pairs, where each of these simulated focal intensity profiles were compared with the 30-degree rotated image. Each of the three focal intensity profiles (“simulated intensity A”) were used as inputs to the forward neural network (which transforms intensity into phase), and the neural network predictions are shown (“predicted phase”). As expected, the predicted phase for the 0-degree intensity profile looks similar to the “current phase”. All three predictions were then used as inputs to the reverse neural network (which transforms phase into intensity) and the neural network predictions are shown (“simulated intensity B”). The “simulated intensity B” intensity profiles for the 0-degree and the 60-degree rotated cases look similar to the associated “simulated intensity A” intensity profiles, hence implying that these two focal intensity profiles are familiar to the neural network and physically possible within this simulation. However, the “simulated intensity B” and the “simulated intensity A” for the 30-degree rotated case look significantly different, hence implying that this focal intensity profile is unfamiliar to the neural network because it is not physically possible within this simulation. The final column (“difference”), which is a subtraction of the simulated intensity A and B images, with green representing a positive difference and red a negative difference, clearly shows that there is little difference for the 0 degree and 60-degree cases, but a significant difference for the 30-degree case.

An important consideration is whether this technique could be scaled up to identify the phases of a larger numbers of fibres, such as 37 or 61 fibres. In our current approach, we have had to balance a range of constraints due to available computational power, and hence we anticipate that this technique could be extended to larger numbers of fibres. Due to diffraction effects, interference between the outer 12 fibres generally produces smaller features in the focal intensity profile than the contributions from the inner 6 fibres. Here, when only illuminating fibres in the outer ring of 12 fibres, the minimum fringe separation in the focal intensity profile was 20 image pixels. However, if the number of fibres was increased, hence resulting in additional rings of fibres, at some point the interference fringes might have a size that approached that of an image pixel, thereby yielding a phase prediction which had a considerably lower accuracy.

To counteract this, there are two options. Firstly, the size of the circles that represent the fibres could be reduced (i.e. a reduction in spatial resolution). For the current arrangement of 19 fibres in a 256 × 256 image, the position, the spatial amplitude, and the phase profile for each fibre is described in ~ 50 × 50 image pixels. If the images were scaled in this way, in order to add more fibres, the number of image pixels used to describe each fibre would be reduced; eventually discretisation effects due to the use of integer values to describe phase and intensity, rather than continuously variable values, and spatial discretisation into pixels would become apparent. Secondly, the size of the array could be increased to enable higher spatial resolution in the focal intensity profiles. In practice, however, an increase in array size from 256 × 256 to 512 × 512 would result in approximately four times the neural network training time. In addition, there would be an increase in time for creating the training data. Whilst increasing the number of fibres would lead to an increase in complexity of the problem that is being solved, and therefore additional training data pairs would almost certainly be required, there is no fundamental limit that we can see to the absolute number of fibres that could be accommodated in such a technique.

To extend this phase prediction approach to 61 fibres for example, each fibre could be described by 28 × 28 image pixels in a 256 × 256 image, by decreasing the spatial resolution of the phase profile image. Importantly, this would result in a minimum fringe separation of ~ 10 image pixels, meaning that information corresponding to the interference of the outer fibre ring would still be present in the simulated intensity profiles, and therefore the neural network should still have information corresponding to the phase of all 61 fibres. While we see that increasing to 37 or 61 fibres, or even beyond, would introduce other challenges, we do not see any a-priori reason why a larger number of fibres could not theoretically be accommodated.

## Conclusions

In conclusion, a method for predicting the phase of nineteen fibres arranged in a hexagonal close-packed array directly from the simulated focal intensity was shown, which has direct application in the optimisation of coherent beam combination. The approach used a conditional generative adversarial network to transform an image of the simulated focal intensity profile into the associated image of the simulated phase profile at the exit of the fibre array. It was shown that subtracting the predicted phase from the current phase would produce a good estimate of a flat phase, which could be used for phase correction or as a basis for adding bespoke phase profiles, and hence, enabling spatial intensity profile control. By training a second neural network to perform the inverse operation, namely the transformation of simulated phase into simulated intensity profile, the two networks could be linked, such that an intensity profile could be transformed into a phase profile and then back to an intensity profile. As, in this simulation, all possible phase profiles lead to an intensity profile, but not all intensity profiles lead to a phase profile, the two networks could be used to identify which intensity profiles were physically possible in this simulation.

## Methods

### Beam propagation simulation

The simulated data was created via the use of the angular spectrum method to propagate electric fields from the fibre exit plane to the focal plane. The simulated electric field was a 1000 × 1000 array, with pixel size of 10 µm, a distance between the fibre plane and the focal plane of 25 cm, a radius of each fibre of 500 µm, and a laser wavelength of 1 µm. The spatial distribution of the electric field amplitude for each fibre was a Gaussian with 1/e^2^ intensity radius as 0.8 of the fibre radius, with a maximum value of one, and with zero amplitude outside the fibre. The phase for each fibre was randomly chosen from a uniform distribution between − π and + π, and the phase for the central fibre was always set as zero. Each random set of phases therefore had an associated focal intensity profile, and hence, both could be used to create a single training (or testing) data pair for the neural network. It was found that a trigonometric representation of the phase was needed to create the phase image, as shown in Fig. 1, where the red channel of the image corresponded to the cosine of the phase, and the blue channel to the sine of the phase. This approach ensured that there was a cyclic change in the colour of the image, rather than a discrete jump from − π to + π. The intensity image was created by making all RGB channels equal to the simulated intensity value, and converted into an 8-bit number (i.e. 0 to 255), and hence acted as a similar quantisation of intensity values to an 8-bit monochrome experimental camera. For each intensity image, the values were normalised to a maximum of 255. Finally, to reduce training time, and hence allow training on a larger number of random phase combinations, the images were reduced to a 256 × 256 resolution. The fibre field was firstly cropped to 512 × 512, and then resized to 256 × 256. The focal intensity was cropped to 100 × 100 and then resized to 256 × 256. The simulated size scales are presented for the two domains in Fig. 1. Due to the computational challenge of creating large numbers of training data pairs needed for this work, a range of high specification personal computers were used (which typically created approximately 1000 training pairs per hour) as well as the IRIDIS High Performance Computing Facility at the University of Southampton.

### Neural network

The network was a conditional generative adversarial network (cGAN), known in the literature as “pix2pix”^41^. This is a well-studied network, capable of learning complex transfer functions between images from two different domains, and hence ideal for learning the complex relationship between an intensity profile and its corresponding reconstructed phase profile. The generator had a U-Net structure, with a downscaling and upscaling path consisting of 8 blocks of 4 × 4 convolutional filters and strides of 2, each followed by a batch normalisation and a leaky ReLU^45^. The downscaling and upscaling paths were connected via concatenation. The discriminator consisted of downscaling using 4 blocks, also consisting of 4 × 4 convolutional filters and strides of 2, each followed by a batch normalisation and a leaky ReLU. A minibatch of size 1 was used, and a generator and discriminator learn rate of 0.0002. The neural network training ran for 1 epoch for all datasets. Training was completed using MATLAB, taking from 2 min (300 epochs) to nearly 36 h (300 k epochs).

## Data Availability

Data supporting this manuscript is available at https://doi.org/10.5258/SOTON/D1982.

## References

[1] Jauregui C, Limpert J, Tünnermann A (2013). High-power fibre lasers. Nat. Photonics.

[2] Canning J (2006). Fibre lasers and related technologies. Opt. Lasers Eng..

[3] Richardson DJ, Nilsson J, Clarkson WA (2010). High power fiber lasers: Current status and future perspectives. JOSA B.

[4] Zervas MN, Codemard CA (2014). High power fiber lasers: A review. IEEE J. Sel. Top. Quantum Electron..

[5] Chraplyvy A, Henry P (1983). Performance degradation due to stimulated Raman scattering in wavelength-division-multiplexed optical-fibre systems. Electron. Lett..

[6] Shiraki K, Ohashi M, Tateda M (1995). Suppression of stimulated Brillouin scattering in a fibre by changing the core radius. Electron. Lett..

[7] Farries M, Rogers A (1983). Temperature dependence of the Kerr effect in a silica optical fibre. Electron. Lett..

[8] He B (2006). High power coherent beam combination from two fiber lasers. Opt. Express.

[9] Kozlov V, Hernandez-Cordero J, Morse T (1999). All-fiber coherent beam combining of fiber lasers. Opt. Lett..

[10] Chang H (2020). First experimental demonstration of coherent beam combining of more than 100 beams. Photonics Res..

[11] Ma P (2021). 7.1 kW coherent beam combining system based on a seven-channel fiber amplifier array. Optics Laser Technol..

[12] Shay TM (2006). Theory of electronically phased coherent beam combination without a reference beam. Opt. Express.

[13] Shay T (2006). First experimental demonstration of self-synchronous phase locking of an optical array. Opt. Express.

[14] Fienup JR (1982). Phase retrieval algorithms: A comparison. Appl. Opt..

[15] Levy, J. L. & Roh, K. Coherent array of 900 semiconductor laser amplifiers. In *Laser Diodes and Applications*, Vol. 2382 (International Society for Optics and Photonics, Bellingham, USA 1995). 10.1117/12.208463.

[16] Zhou P (2009). Coherent beam combining of fiber amplifiers using stochastic parallel gradient descent algorithm and its application. IEEE J. Sel. Top. Quantum Electron..

[17] Vorontsov MA, Sivokon VP (1998). Stochastic parallel-gradient-descent technique for high-resolution wave-front phase-distortion correction. JOSA A.

[18] Kokhanovskiy A (2019). Machine learning methods for control of fibre lasers with double gain nonlinear loop mirror. Sci. Rep..

[19] Närhi M (2018). Machine learning analysis of extreme events in optical fibre modulation instability. Nat. Commun..

[20] Mills B (2018). Predictive capabilities for laser machining via a neural network. Opt. Express.

[21] Heath DJ (2018). Machine learning for 3D simulated visualization of laser machining. Opt. Express.

[22] Mills B, Grant-Jacob JA (2021). Lasers that Learn: The Interface of Laser Machining and Machine Learning.

[23] Sonoda S, Murata N (2017). Neural network with unbounded activation functions is universal approximator. Appl. Comput. Harmon. Anal..

[24] Sinha A (2017). Lensless computational imaging through deep learning. Optica.

[25] Grant-Jacob JA (2020). Lensless imaging of pollen grains at three-wavelengths using deep learning. Environ. Res. Commun..

[26] Song J (2020). Coherent beam combining based on the SPGD algorithm with a momentum term. Optik.

[27] Hou T (2019). High-power vortex beam generation enabled by a phased beam array fed at the nonfocal-plane. Opt. Express.

[28] Chang Q (2021). Iteration-free, simultaneous correction of piston and tilt distortions in large-scale coherent beam combination systems. Opt. Express.

[29] Wang D (2021). Stabilization of the 81-channel coherent beam combination using machine learning. Opt. Express.

[30] Hou T (2020). Deep-learning-based phase control method for tiled aperture coherent beam combining systems. High Power Laser Sci. Eng..

[31] Hou T (2020). Deep-learning-assisted, two-stage phase control method for high-power mode-programmable orbital angular momentum beam generation. Photonics Res..

[32] Tünnermann H, Shirakawa A (2019). Deep reinforcement learning for coherent beam combining applications. Opt. Express.

[33] Zhang X (2021). Coherent beam combination based on Q-learning algorithm. Opt. Commun..

[34] Tünnermann H, Shirakawa A (2021). Deep reinforcement learning for tiled aperture beam combining in a simulated environment. J. Phys. Photonics.

[35] Soulard R (2014). ICAN: A novel laser architecture for space debris removal. Acta Astronaut..

[36] Mourou G (2013). The future is fibre accelerators. Nat. Photonics.

[37] Dickey, F. M., Weichman, L. S. & Shagam, R. N. Laser beam shaping techniques. In *Proc. SPIE 4065, High-Power Laser Ablation III*. 10.1117/12.407361 (International Society for Optics and Photonics, 2000).

[38] Adamov E (2021). Laser beam shaping based on amplitude-phase control of a fiber laser array. OSA Contin..

[39] Hou T (2020). Comprehensive investigation on producing high-power orbital angular momentum beams by coherent combining technology. High Power Laser Sci. Eng..

[40] Prieto C (2020). Dynamic laser beam shaping for laser aluminium welding in e-mobility applications. Procedia CIRP.

[41] Isola, P., et al. Image-to-image translation with conditional adversarial networks. In *Proceedings of the IEEE Conference on Computer Vision and Pattern Recognition* (2017).

[42] Matsushima K, Shimobaba T (2009). Band-limited angular spectrum method for numerical simulation of free-space propagation in far and near fields. Opt. Express.

[43] Jabczyński JK (2021). Simplified sensitivity analysis of coherent beam combining in a tiled aperture architecture. Appl. Opt..

[44] Jabczynski JK, Gontar P (2019). Effect of beam profile and partial coherence on coherent beam combining performance. Opt. Commun..

[45] Agarap, A. F. *Deep Learning Using Rectified Linear Units (relu).* arXiv preprint http://arxiv.org/abs/1803.08375 (2018).

